# An assessment of optimizing biofuel yield percentage using K-fold integrated machine learning models for a sustainable future

**DOI:** 10.1371/journal.pone.0328880

**Published:** 2025-08-14

**Authors:** Krishnamoorthy Ramalingam, Mohd. Zulkifly Abdullah, Perumal Venkatesan Elumalai, Allam Sangeetha, Xu Yong, Nasim Hasan, Wei Shangzhi

**Affiliations:** 1 School of Mechanical Engineering, Engineering campus, Universiti Sains Malaysia, Nibong Tebal, Penang, Malaysia; 2 Department of Sustainable Energy Engineering, Saveetha School of Engineering, Saveetha Institute of Medical and Technical Science, Chennai, India; 3 Department of Mechanical Engineering, Aditya University, Surampalem, India; 4 Department of Computer Science and Engineering, MLR Institute of Technology, Hyderabad, Telangana, India; 5 School of Artificial Intelligence and Smart Manufacturing, Hechi University, Yizhou, China; 6 Mettu University, Metu, Ethophia; 7 Faculty of Education, Shinawatra University, Bang Toei, Thailand; Shahrekord University, IRAN, ISLAMIC REPUBLIC OF

## Abstract

Accelerating population and modernization has triggered a steady rise in energy demand and a significant rise in household waste, particularly municipal solid waste. In this context, waste-to-energy conversion has emerged as a sustainable solution. This study aims to maximize biofuel production yield using biomass-based banana peel catalyst waste by optimizing process parameters through machine learning models integrated with k-fold cross-validation. The models employed include Polynomial Regression (PR), Decision Tree (DT), Random Forest (RF), and Linear Regression (LR). The three key input variables including reaction temperature (RT), catalyst concentration (CC), and methanol-to-oil molar ratio (MOR) were used to train and test the models, with biodiesel yield as the measured output. Among the models, PR emerged as the best-performing one for predicting biofuel yield, demonstrated by its high R² value of 0.956 and low error metrics (RMSE = 1.54 MSE = 2.39 MAE = 1.43). The best model was determined through balancing bias and variance across k-fold validation iterations, where PR exhibited the highest average R² value of 0.868. Furthermore, the optimized process parameters predicted by PR for maximum biofuel yield were a RT of 59°C, CC of 2.96%, and a MOR of 9.21, resulting in a yield of 95.38%. These findings contribute to advancing large-scale machine learning-driven biofuel optimization, supporting industrial waste-to-energy applications, and fostering sustainable energy development.

## Introduction

Waste-to-energy conversion has emerged as a widely recognized strategy for promoting sustainability to addressing the dual challenges of biomass disposal and fossil fuel dependence. Surging global population growth combined with modernization has intensified global energy consumption, causing several countries to rely extensively on fossil fuels for transportation and electricity generation. However, fossil fuels are non-renewable, hydrocarbon-based energy sources that pose environmental and health risks while becoming progressively costlier due to depletion [1–3]. In response, researchers worldwide are exploring alternative energy solutions, including electric vehicles (EVs), hydrogen-based fuels, and biofuels derived from agricultural and biomass sources [2,4].

Among these renewable energy options, biofuels have emerged as a promising alternative, offering scalable solutions for sustainable energy production while addressing the limitations of EVs and hydrogen fuels. EV adoption is hindered by inadequate charging infrastructure, battery reliability concerns, and e-waste management challenges. Meanwhile, hydrogen-based fuels, particularly blue hydrogen, present environmental risks and storage complications, reducing their practicality in widespread energy applications. Given these constraints, biomass- and agriculture-based biofuels have gained prominence due to their efficient conversion processes and compatibility with existing fuel systems [5–8].

A critical factor in biofuel production is the selection of suitable feedstocks, which must meet criteria such as availability, cost-effectiveness, renewability, and non-edibility [9,10]. Seed-based feedstocks are commonly chosen for their high biodiesel yield; however, their elevated viscosity and fatty acid content pose challenges for combustion efficiency. To address these limitations, fuel upgrading processes have been developed, with transesterification standing out as one of the most effective techniques [11,12].

Transesterification has demonstrated superior thermal properties compared to other fuel upgrading techniques such as water emulsion, blending, and pyrolysis. However, biofuel yield is highly dependent on key reaction parameters namely RT, CC, MOR, and reaction duration, which directly affect conversion efficiency [13–15]. Researchers have investigated diverse catalysts utilized to facilitate transesterification, including homogeneous, heterogeneous, enzymatic, and supercritical alcohol-based catalysts. Among them, alkaline catalysts such as NaOH and KOH are commonly used for their high reactivity and fast conversion rates, particularly with low free fatty acid feedstocks. Acid catalysts perform better for feedstocks with high FFA content despite their slower reaction kinetics, while heterogeneous catalysts offer sustainability benefits through reusability and improved separation processes. Supercritical transesterification provides an alternative method, achieving high biofuel yields without catalyst contamination [16–18].

Many researchers have favored heterogeneous catalysts in biodiesel synthesis for their reusability, sustainability, and ease of separation from the final product. Unlike homogeneous catalysts, which require additional purification steps, heterogeneous catalysts enable multiple reaction cycles, reducing waste and improving process efficiency [19,20]. Among the widely studied heterogeneous catalysts, metal oxides such as CaO, MgO, ZnO, TiO₂ and etc have demonstrated high catalytic activity and stability. Table 1 provides an extensive survey on the application of heterogeneous catalysts in biodiesel synthesis, highlighting their effectiveness across different feedstocks and optimization techniques. Such catalysts persist as key enablers in the evolution of biofuel research, contributing to sustainable energy advancements.

**Table 1:** Summary of Heterogeneous Catalysts Used in Biodiesel Production Along with Influential Process Parameter.

S.No	Feedstock	Heterogeneous Catalyst	Optimum Catalyst Concentration (wt%)	Optimum Reaction Temperature (°C)	Optimum Methanol-to-Oil Molar Ratio	Optimum Reaction Duration (min)	Optimum Biodiesel Yield (%)	Most Influential Parameter
1	Waste Cooking Oil [21]	CaO	5	65	12:1	120	96.47	Catalyst Concentration
2	Jatropha Oil [22]	MgO	7.5	60	10:1	50	92.3	Reaction Temperature
3	Soybean Oil [23]	ZnO based composite catalyst	4	65	9:1	15	97%	Methanol-to-Oil Ratio
4	Palm Oil [24]	Calcareous egg shell based catalyst	7	150	12:1	180	94.5	Catalyst Concentration
5	Microalgae Oil [25]	Fe3o4@SiO₂	5	60	13:1	55	97%	Methanol-to-Oil Ratio
6	Sunflower Oil [26]	K₂CO₃	4	120	15:1	50	89	Reaction Temperature
7	Castor Oil [27]	Zn/Mg	3	55	9:1	40	87	Catalyst Concentration
8	Cottonseed Oil [28]	KoH	6	55	6:1	60	96	Methanol-to-Oil Ratio
9	Waste chicken fat [29]	eggshell	2	65	10:1	55	90.2	Catalyst Concentration

Traditionally, process optimization in biodiesel production has relied on design of experiments (DoE) techniques such as Taguchi, ANOVA, and Response Surface Methodology (RSM) to identify ideal parameter combinations. Although these statistical methods provide valuable insights, they often struggle to capture nonlinear interactions among multiple variables, resulting in prediction errors ranging from 4–5%. To enhance predictive accuracy and optimize process parameters more effectively, machine learning (ML) techniques have recently gained prominence [30–32].

Machine learning models including ANN, PR, SVR, DT, RF, and KNN offer refined approaches for biodiesel yield prediction, outperforming conventional statistical methods in terms of reliability and accuracy [33,34]. To further improve prediction efficiency, K-fold cross-validation is integrated into ML models, ensuring robust evaluation and generalization. By segmenting the dataset into distinct subsets in a systematic manner and iteratively training the model, K-fold validation reduces bias and mitigates overfitting, making the selected model more reliable for process optimization [35,36].

Based on the literature survey, minimal research has discovered the application of machine learning models in predicting biodiesel yield using previously published datasets, particularly in the context of biomass-based heterogeneous catalysts. Therefore, this study employs four machine learning models Polynomial Regression (PR), Linear Regression (LR), Decision Tree (DT), and Random Forest (RF) to analyze and optimize process parameters for biodiesel production. These models are integrated with K-fold cross-validation to improve prediction accuracy and enhance model reliability. Additionally, SHAP factor analysis and heatmap visualization are conducted to identify the most influential parameters affecting biodiesel yield, providing deeper insights into process optimization based on existing experimental data.

## Material and methodology

### Catalyst preparation

Biomass-based banana peel-derived catalyst was prepared using agro-waste collected from local fruit vendors, restaurants, and vegetable markets. Initially, the gathered banana peels underwent thorough washing using distilled water to ensure the removal of surface contaminants and residual dirt. Any residual fruit material was manually separated and discarded. The cleaned peels were then sundried to reduce surface moisture and cut into smaller pieces for uniform drying. Subsequently, the chopped peels were oven subjected to drying at 60°C for two hours to remove remaining moisture content. The oven-dried biomass was subsequently calcined at 900°C to induce complete decomposition of organic matter and conversion into a carbon-rich calcinated catalyst. After calcination, the solid product was finely milled and preserved in a sealed container to prevent moisture absorption.

### Procedure for biodiesel production

The transesterification of the composite oil blend comprising 20% neem oil and 80% used cooking oil was performed using a heterogeneous base catalyst synthesized from calcinated banana peels, as outlined in Section 3.1. The biodiesel production process began with the pre-treatment to eliminate impurities and reduce moisture content. The composite oil was initially filtered and preheated to facilitate the removal of water, which can otherwise impede catalyst performance and encourage soap formation. Due to the elevated FFA content in the composite oil, an acid-catalyzed esterification step was carried out using sulfuric acid to lower the FFA concentration to acceptable levels. In this process, in this process, 20 vol% methanol and 1 wt% H₂SO₄ were added to the pretreated oil and stirred at 70°C under controlled conditions to convert FFAs into methyl esters while minimizing the risk of saponification. Upon completion of the reaction, the mixture was neutralized and Rinsed thoroughly using distilled water to remove any remaining acid and byproducts, preparing the composite oil for the subsequent transesterification step.

**Fig 1:**
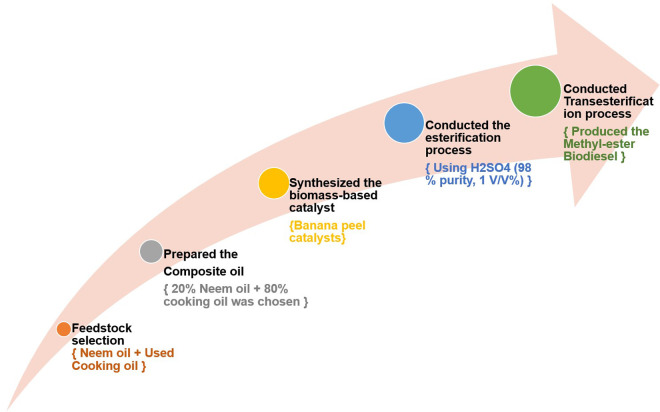
The schematic representation of steps involved in biodiesel production.

The transesterification was conducted in a three-necked round-bottom flask fitted with a mechanical stirrer and digital thermometer to maintain thermal stability. Transesterification experiments were carried out across a range of parameters: methanol-to-oil molar ratio (6–12:1), catalyst loading (1–3 wt% of oil), and reaction temperature (55–65°C), with maintained a fixed reaction duration of 60 minutes with continuous stirring at 600 rpm. At the beginning of each run, the required amount of catalyst was dispersed in methanol and stirred for 10 minutes to ensure uniform mixing before being introduced into the reactor containing the pre-heated composite oil. The reaction mixture was kept under constant agitation throughout to promote effective mass transfer and maintain homogeneity. Upon completion, the mixture was poured into a separating funnel and allowed to stand for 12 hours to enable phase separation under gravity. Two distinct layers formed: an upper biodiesel-rich layer and a lower glycerol-rich layer. The biodiesel phase was carefully decanted, washed repeatedly using heated distilled water to ensure complete removal of catalyst residues, soap, and methanol, and subsequently dried in a hot air oven at 105°C for two hours to ensure complete moisture removal. The end product was securely stored in closed containers for further characterization and analysis [37]. A schematic depiction of the biodiesel production steps is provided in Fig 1, highlighting both the esterification and transesterification stages.

### Background of ML regression model

In machine learning applications for biodiesel production, researchers frequently utilize regression models, classifiers, and ANN to refine experimental parameters to achieve optimal outcomes. For this study, regression models were selected based on pragmatic factors such as data availability, computational efficiency, and their appropriateness for the intended research objectives. These models encompass a diverse range of algorithms designed to accommodate specific data structures and predictive requirements, ensuring robust and interpretable results [38,39].

**Fig 2:**
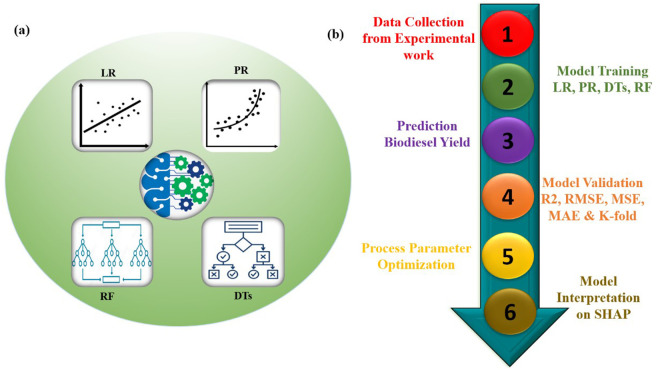
Overview of ML work flow a) ML models b) steps involve in work flow.

To effectively predict and validate experimental results in biodiesel production, four regression algorithms were employed, each offering distinct advantages in handling diverse data patterns. Linear Regression (LR) is well-suited for capturing straightforward relationships between variables, making it ideal for modeling linear trends. In contrast, Polynomial Regression (PR) accommodates more complex, non-linear interactions, providing greater flexibility in identifying intricate patterns within the dataset. Decision Trees (DT) offer interpretable models for complex datasets, while Random Forests (RF) enhance prediction accuracy and mitigate overfitting by aggregating multiple decision trees [40,41]. The full machine learning workflow is presented in Fig 2.

Hyperparameters significantly influence how machine learning models learn and generalize to unseen data. To ensure learning accuracy, we have selected suitable hyper parameters of the models through a trial-and-error approach the selected hyperparameters were provided in Table 2. The intercept and normalization are key parameters in LR and degree of the polynomial is the key parameter in PR. Additionally, min_samples_leaf in DTs and n_estimators in RF are vital parameters to improve model stability and balance the underfitting and overfitting effectively. These adjustments ensure that each model learns meaningfully from the dataset while mitigating performance biases.

**Table 2:** Hyper Parameters for all four ML learning models.

Sl. No	ML Models	Hyper Parameters
1	LR	Linear regression with intercept enabled and normalization turned off.
2	PR	PR of degree 2 without bias term.
3	DTs	No limit on tree depth, splitting requires 2 samples, each leaf must have 1 sample, and no feature limit.
4	RF	Random Forest with 10 trees, unlimited depth, minimum 2 samples to split, leaf size 1, feature selection is automatic, with bootstrapping and fixed seed of 42.

### Data collection

The L27 orthogonal array design was employed as the Design of Experiments (DOE) methodology to systematically generate experimental data, as illustrated in Table 3. The dataset used in this study originates from a previously published work [37]. The dataset consists of 27 entries, partitioned into 80% for training and 20% for testing to support robust predictive modeling. The selected input parameters include reaction temperature (RT), catalyst concentration (CC), and methanol-to-oil molar ratio (MOR), while the output parameter being assessed is biodiesel yield.

**Table 3:** Experimental Design Matrix Based on L27 Orthogonal Array for Process Parameter Optimization.

SI. No	Reaction Temperature (RT)	Methanol to oil molar ratio (MOR)	Catalyst concentration (CC)	Experimental Yield
1	55	9	1	60.14
2	55	12	1	78.33
3	60	9	1	76.33
4	60	12	1	74.34
5	60	6	1	72.38
6	65	9	1	73.42
7	65	12	1	72.41
8	65	6	1	69.33
9	55	9	2	91.54
10	55	12	2	91.61
11	55	6	2	89.14
12	60	9	2	91.52
13	60	12	2	91.24
14	60	6	2	88.44
15	65	9	2	87.65
16	65	12	2	87.45
17	65	6	2	85.31
18	55	9	3	94.25
19	55	12	3	94.57
20	55	6	3	94.33
21	60	9	3	94.18
22	60	12	3	94.35
23	60	6	3	94.51
24	65	9	3	94.14
25	65	12	3	94.21
26	65	6	3	94.35
27	55	9	1	60.14

**Fig 3:**
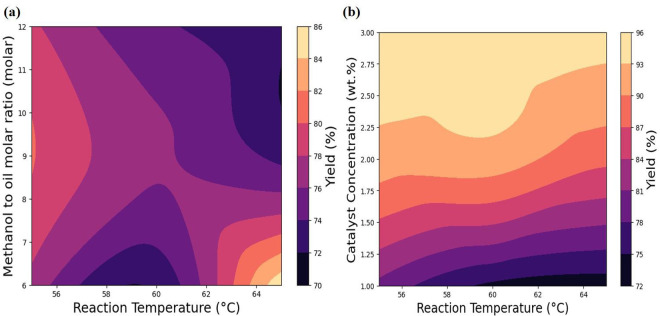
Visualization of Experimental data collection a) Reaction Temperature, Methanol to molar ratio verus biodiesel yield b) Reaction Temperature, Catalyst concentration versus biodiesel yield.

The dataset spans a structured range, with RT varying between 55°C and 65°C, CC from 1 wt% to 3 wt%, and MOR extending from 6 to 12. These key variables are visually presented using contour plots in Figs 3a and 3b, facilitating a comprehensive understanding of data distribution. Furthermore, Fig 3c provides a statistical summary, offering insights into overall trends and significant characteristics within the dataset. For ML prediction, Python Google Colab (Packaged as version 1.0.0 and compliant with Apache License, v2.0 terms) was used to build and train the model, utilizing the collected data from previous work along with Scikit-learn library.

### K-fold cross validation

Traditional regression models typically utilize an 80:20 holdout validation, where the dataset was split into training (80%) and testing (20%) subsets to assess model performance. Performance is assessed through R² and error metrics, with higher R² and lower error indicating better predictive accuracy. However, this single-split approach can introduce biases due to uneven data partitioning.

**Fig 4:**
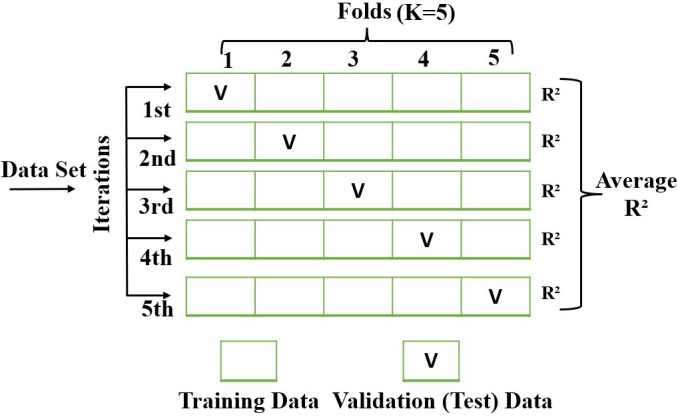
Basic concept of five k-fold cross-validation.

To enhance reliability, Five k-fold cross-validation was employed, partitioning the dataset into five equally sized subsets. The model undergoes five iterative cycles, systematically using each fold as a test set while training on the remaining folds. This ensures comprehensive data utilization and mitigates overfitting risks. Averaging the five R² values obtained across iterations provides a more robust and unbiased model evaluation, particularly beneficial for small datasets [42,43]. The methodology of five k-fold cross-validation is schematically represented in Fig 4, demonstrating its role in improving process parameter predictions for optimizing biodiesel yield.

## Results and interpretations

### ML Learning model prediction on biodiesel yield

**Fig 5:**
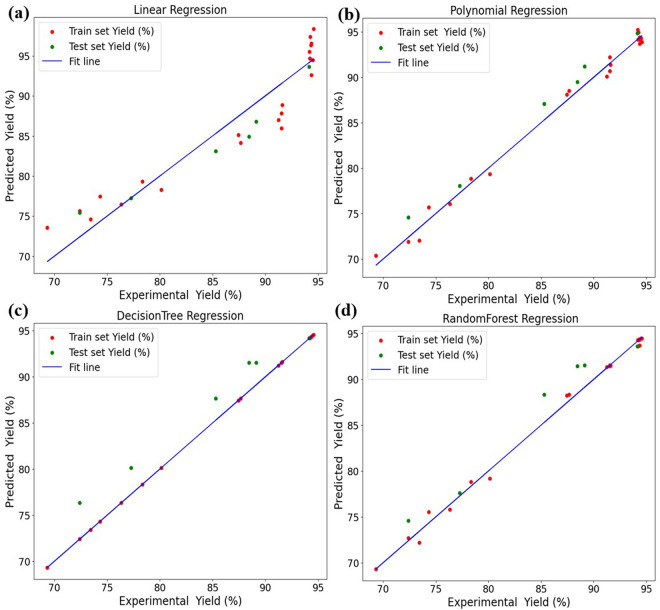
Comparison of experimental output verus prediction output by all four learning models a) LR, b) PR, c) DTs, d) RF.

All four selected machine learning algorithms were utilized for forecasting biodiesel yield based on investigational data, with results illustrated in Fig 5. The comparison between predicted yield and experimental yield was visualized using fit lines, test points, and train points, enabling an assessment of each model’s accuracy in replicating real-world trends. From this evaluation, Polynomial Regression (PR) and Random Forest (RF) exhibited the highest similarity to experimental outputs, demonstrating their effectiveness in capturing complex interactions within the dataset. Further comparison revealed that PR and RF exhibited strong alignment with the fit line and minimal deviation from test and train points, reinforcing their reliability in accurately predicting biodiesel yield across varying process parameters. This robust predictive capability highlights their potential for optimizing biodiesel production efficiency.

The selected machine learning models were further validated using critical evaluation indicators, including the R² and error values such as RMSE, MSE, and MAE. In general, an elevated R² combined with low error values signifies superior predictive accuracy [44]. Thus, models demonstrating strong correlation between predicted and experimental outcomes were considered the most reliable for biodiesel yield prediction.

**Fig 6:**
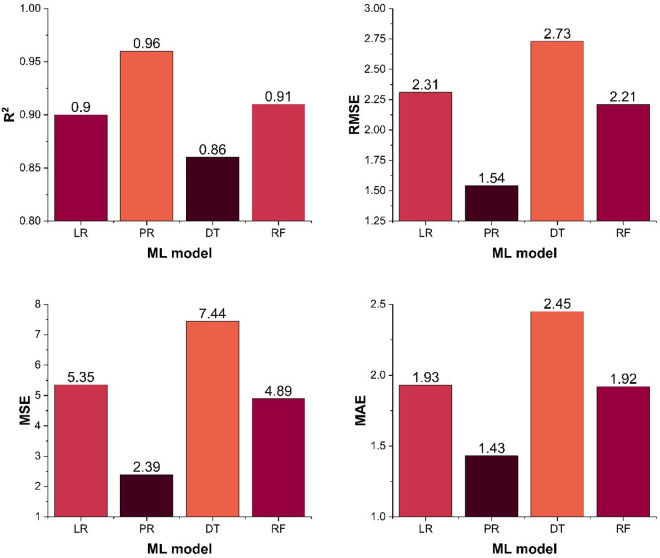
Validation metric for all four learning models a) R2, b) RMSE, c) MSE, d) MAE.

Fig 6 provides a detailed visual representation of these evaluation metrics across all models. The assessment confirmed that PR and RF unveiled the highest reliability, with R² values of 0.956 for PR and 0.911 for RF, indicating strong alignment with actual experimental results. Furthermore, the error values for PR and RF models were RMSE = 1.54, MSE = 2.39, MAE = 1.43 for PR, and RMSE = 2.21, MSE = 4.89, MAE = 1.92 for RF. These results highlight the robustness of PR and RF, reinforcing their effectiveness in accurately predicting biodiesel yield across various process parameters. Additionally, the reliability valuation emphasizes the significance of model selection and performance evaluation in optimizing biodiesel production processes.

### Report on K-fold validation

**Fig 7:**
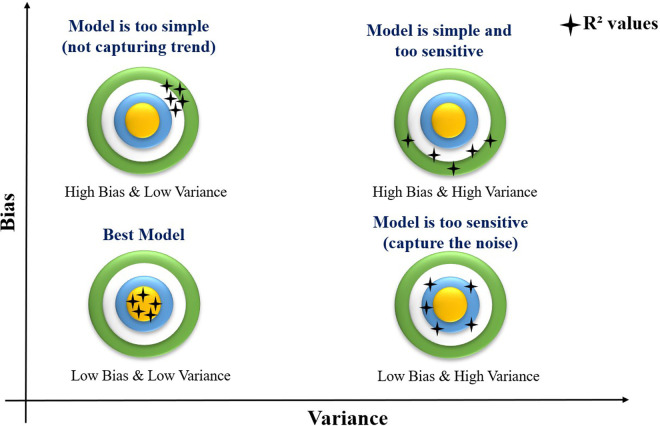
Concept of bias-variance tradeoff for all learning model.

K-fold cross-validation is a key technique for evaluating machine learning model performance via assessment of bias and variance. The interplay between bias and variance is graphically represented in Fig 7, classifies models into four distinct categories: optimal models, overly simple models, overly complex models, and unstable models. These distinctions are based on R² values, which serve as essential indicators of prediction accuracy. An overly simple model exhibits high bias but low variance, leading to generalization errors due to insufficient learning. Conversely, a high-bias, high-variance model struggles to identify patterns, making predictions unreliable. A low-bias, high-variance model captures trends but is highly sensitive to noise, resulting in inconsistent outputs. In contrast, an optimal model achieves low bias and low variance, ensuring stable and accurate predictions across diverse datasets. By systematically evaluating bias and variance, k-fold cross-validation enhances model reliability, minimizes errors, and optimizes parameter selection for better generalization in machine learning applications [45,46].

From the five-fold cross-validation, a total of 20 R² values were obtained, with each learning algorithm contributing five high R² values. These values were averaged to determine the mean R² score for each model, as tabulated in Table 4. The tabulated results specify that the PR model attained the highest average R² value of 0.868, surpassing all other models. Similarly, the RF model recorded the second-highest average R² value of 0.836, demonstrating strong predictive reliability.

**Table 4:** K-fold cross validation on average R^2^ results.

Iterations (k = 5)	R2 values for ML models			
	LR	PR	DTs	RF
**Iteration 1**	0.76	0.91	0.98	0.8
**Iteration 2**	0.91	0.91	0.97	0.99
**Iteration 3**	0.81	0.89	0.75	0.96
**Iteration 4**	−1.13	0.72	0.93	0.78
**Iteration 5**	0.87	0.91	0.54	0.65
**Average R** ^**2**^	0.444	0.868	0.834	0.836

Both PR and RF fall under the category of low bias and low variance, meaning they consistently yield predictions closer to the ideal value of 1 across all iterations. Their stable performance underscores their suitability for accurately modeling biodiesel yield prediction, reinforcing their efficacy in identifying nonlinear trends and interactions.

### ML learning model interpretation

**Fig 8:**
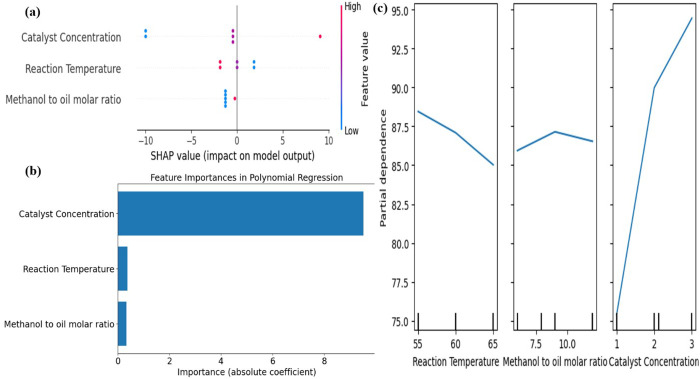
model interpretation a) SHAP b) Feature Importance c) Partial dependence.

For model interpretation, SHAP values analysis, Feature importance, Partial dependence and Pearson correlation heatmap were employed to evaluate the best-performing learning algorithm, as determined via five k-fold cross-validation. The Polynomial Regression (PR) model demonstrated the highest prediction accuracy, closely aligning with experimental results. Consequently, the PR model underwent SHAP values analysis providing deeper insights into the influence of input variables on biodiesel yield predictions. The Feature importance and Partial dependence analysis provide quantities significant influence and trend of input variables. The Heatmap analysis used to provide liner corelation of input and output variables [47,48]. The Fig (8a) presents the relationship between SHAP values and feature inputs for the Polynomial Regression (PR) model, offering an in-depth interpretation of how individual parameters influence predictions.

The SHAP visualization follows a structured format, where wider sections highlight features that exert significant impact on the output response. In a SHAP summary plot, each dot denotes a prediction with its color, red for high, blue for low, and purple for medium values. The spread of dots for a given feature reflects its impact on predictions, with wider distributions signifying stronger influence. Features are ranked top to bottom in order of decreasing importance. An optimistic SHAP value signifies a feature’s positive correlation with the output, A negative SHAP value implies that the feature contributes to lowering the predicted response. The SHAP value representation highlights catalyst concentration as a key determinant in biodiesel yield prediction, with its wider sections representing a substantial influence on the output. Furthermore, the feature value transition from blue to red suggests a positive correlation, implying that increasing the catalyst amount promotes greater biodiesel production within the range of 1–3% of catalyst.

The analysis presented in Fig (8b) indicates that catalyst concentration holds significant feature importance in biodiesel yield prediction, with a magnitude coefficient nearing 10. In contrast, RT and MOR exhibit considerably lower influence, with magnitude coefficients reaching only 0.5. This suggests that catalyst concentration accounts for approximately 95% of the impact on biodiesel yield, emphasizing its dominant role in process optimization.

The partial dependence analysis illustrates the influence trends of input variables on the output. Fig (8c) shows that catalyst concentration has a continuously increasing effect on yield, with higher concentrations (within the range of 1% to 3%) leading to greater biodiesel yield. In contrast, the reaction temperature exhibits an inverse trend, where an increase in temperature (from 55^o^C to 65^o^C) results in a continuous decrease in yield. Meanwhile, the MOR initially enhances biodiesel yield as methanol concentration increases within a certain range. However, beyond this optimal range, the trend reverses, leading to a decline in yield.

**Fig 9:**
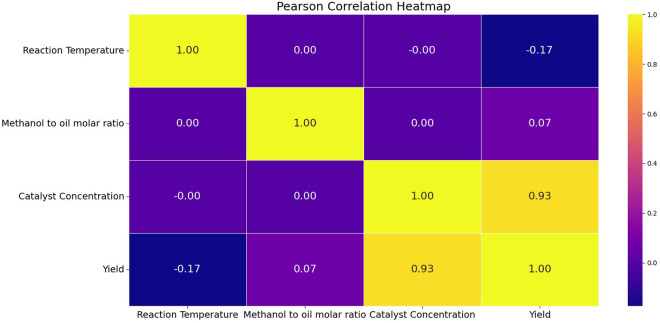
Model interpretation on Heatmap.

The heatmap reveals three distinct relational patterns: positive, negative, and null correlations, with coefficient values ranging from –1 to +1. A coefficient nearing +1 denotes a direct relationship, where increasing input constraints correspond to a rise in output constraints. In contrast, values approaching –1 reflect an inverse relationship, indicating that higher input constraints lead to reduced output constraints. A coefficient near zero suggests no discernible linear association between the input and output variables [45,46]. Fig 9 highlights that catalyst concentration exhibits a strong relational pattern with biodiesel yield, as evidenced by a coefficient of 0.93. This recommends that greater catalyst concentrations lead to an enhanced biodiesel yield, reinforcing its significance in optimizing production efficiency

### ML based optimization output by the best model

The optimization analyses were conducted by ML based PR learning model for finding the optimum process parameters for maximizing the biodiesel yield and it was visualized by two approaches namely 2D and the 3D by adapting PR model. In the 2D, one input variable was varied while the other two were kept constant, allowing for an isolated assessment of the impact of the selected variable on biodiesel yield. Conversely, in the 3D, two input variables were varied while keeping one constant, enabling a more comprehensive interaction analysis among multiple factors.

**Fig 10:**
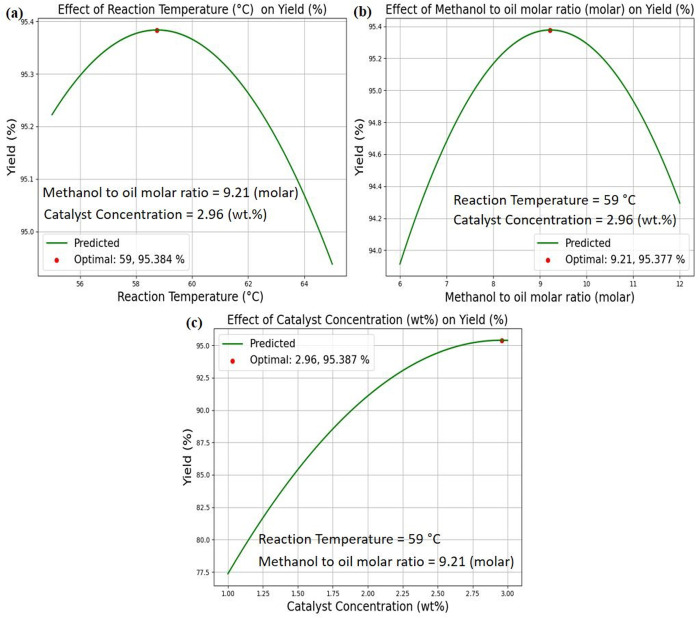
Optimum process parameters by best model a) Reaction Temperature (RT) b) Methanol to oil molar ratio (MOR), c) catalyst concentration (CC).

The optimization results obtained using the 2D are presented in Fig 10, illustrating the impact of different process parameters on biodiesel yield. In Fig 10a, the reaction temperature (RT) was varied while catalyst concentration (CC) and methanol-to-oil molar ratio (MOR) remained constant. The analysis determined that the optimum reaction temperature for achieving maximum biodiesel yield was 59°C, leading to a biodiesel yield of 95.38%.

The results indicate a steady increase in biodiesel yield from 55°C, reaching an optimal level at 59°C. However, beyond this temperature, the yield begins to decline, suggesting an upper threshold where excessive thermal effects negatively impact reaction kinetics. This decline may be attributed to increased feedstock solubility at higher temperatures, potentially triggering side reactions that reduce overall efficiency. These findings align with previous research, which reports that beyond a certain temperature limit, increased feedstock solubility can lead to unintended reactions affecting biodiesel production efficiency [49,50].

Similarly, Fig 10b illustrates the optimization results for the MOR, keeping CC and RT constant. The study identified an optimum ratio of 9.21, achieving a maximum biodiesel yield of 95.38%. The results indicate a progressive increase in yield starting from a ratio of 6, but beyond 9.21, the yield declined. This decrease is likely due to excess methanol disrupting phase equilibrium, which negatively affects biodiesel conversion efficiency. Additionally, an excessive methanol ratio may promote unwanted by-product formation, complicate separation and lowering the overall biodiesel yield These findings align with previous research, which reports that exceeding an optimal methanol ratio can lead to reduced conversion efficiency and increased by-product formation [51,52].

Furthermore, Fig 10c presents the optimization analysis for CC, with MOR and RT held constant. The results confirmed that an optimum CC of 2.96% produced the highest biodiesel yield of 95.38%. The yield increased consistently from 1%, but beyond 2.96%, the reaction may reach a saturation point where additional catalyst no longer enhances conversion efficiency. Excess catalyst could lead to agglomeration or increased viscosity in the reaction mixture, reducing mass transfer efficiency and limiting further yield improvements. These findings align with previous research, which reports that exceeding the optimal catalyst concentration can result in diminished conversion rates due to viscosity changes and agglomeration effects, ultimately affecting biodiesel yield efficiency [53,54].

**Fig 11:**
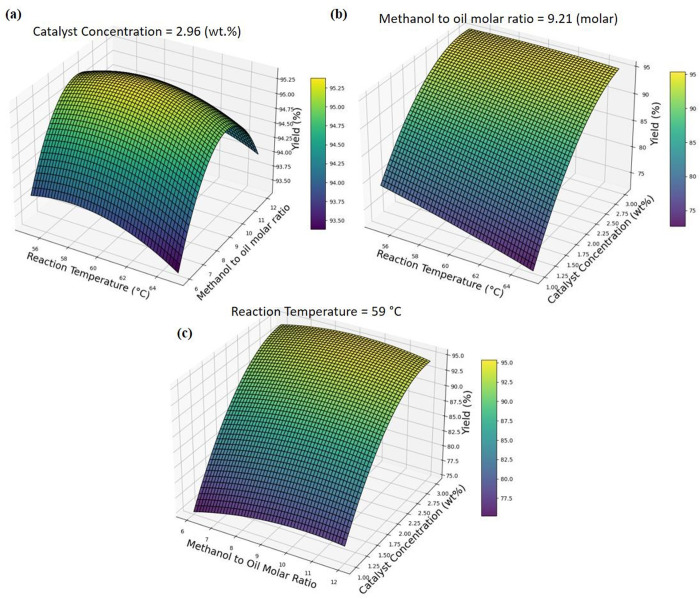
3D illustration of optimum process parameters on maximum yield a) Reaction temperature (RT) versus Methanol to oil molar ratio (MOR), b) Reaction temperature (RT) versus catalyst concentration (CC), c) Methanol to oil molar ratio (MOR) versus catalyst concentration (CC).

The optimization results obtained using the 3D surface plot in Fig 11a illustrate the interactive effects of RT and MOR on biodiesel yield, with CC held constant. The surface reveals a nonlinear synergistic relationship between these two parameters: increasing the RT enhances the transesterification rate by accelerating molecular interactions, with the optimum observed at 59°C. Concurrently, raising the MOR shifts the reaction equilibrium toward biodiesel formation, with the optimum at 9.21. However, beyond these thresholds, the biodiesel yield begins to decline slightly, which can be attributed to excess methanol lowering the reactant concentration per unit volume (dilution effect), causing inefficient mixing and phase separation, and elevated temperatures accelerating methanol evaporation, which reduces the actual methanol available for the reaction. The curved response surface suggests that neither parameter alone maximizes yield; rather, their combined balance leads to the peak biodiesel yield of 95.38%. These findings align with previous research, which reports that exceeding certain thresholds such as RT above 60°C and MOR beyond approximately 9:1 can lead to unintended side reactions and phase imbalance due to increased feedstock solubility and reagent dilution, ultimately compromising biodiesel production efficiency [55–57].

The Fig 11b illustrate the interactive effects of RT and CC on biodiesel yield, with the MOR held constant. The surface reveals a nonlinear coupled relationship between these two parameters: increasing the reaction temperature enhances transesterification kinetics by lowering activation energy barriers and promoting more rapid molecular collisions, with the optimal yield observed at 59°C. Simultaneously, increasing the CC provides more active sites for the reaction, effectively accelerating the conversion of triglycerides to methyl esters. However, beyond the optimal catalyst loading of 2.96%, the biodiesel production exhibits a downward trend. This drop is likely due to catalyst agglomeration or excessive base availability, which can promote saponification reactions and result in soap formation, thereby hindering mass transfer, increasing viscosity, and complicating product separation. The curvature of the response surface demonstrates that a balanced combination of both elevated temperature and adequate catalyst concentration is required to achieve the highest yield, rather than the independent maximization of either factor. These findings are consistent with prior studies that report excessive catalyst dosages and high thermal input can introduce undesired side reactions and separation difficulties, ultimately reducing conversion efficiency and final product purity [53,58].

The Fig 11c illustrate the interactive effects of MOR and CC on biodiesel yield, with the RT held constant. The response surface reveals a nonlinear coupled relationship between these two parameters: increasing the MOR initially drives the transesterification reaction forward enhancing biodiesel conversion. Similarly, increasing the catalyst concentration introduces additional active sites, accelerating the reaction rate and supporting more efficient breakdown of triglycerides into methyl esters. However, beyond the optimal point identified near 9.21 molar ratio and 2.96% catalyst concentration the yield begins to plateau or decline. This behavior is attributed to methanol oversaturation, which can dilute reactant concentration, interfere with phase separation, and reduce the mass transfer rate. Concurrently, excessive catalyst can increase system viscosity or promote particle agglomeration and soap formation, all of which hinder effective mixing and limit reaction efficiency. The curvature of the response surface confirms that biodiesel yield is maximized only when both parameters are simultaneously optimized, rather than altered independently. These observations are consistent with prior research indicating that overloading either methanol or catalyst beyond their critical thresholds can lead to undesirable reaction conditions and reduced conversion performance [59–61]. From the optimizing analysis using PR model the optimised process parameters were founded using polynomial regression equation and it was mentioned in Table 5.

**Table 5:** Optimum process parameters for maximum biodiesel yield.

Reaction Temperature (RT)	Methanal to oil Molar ratio (MOR)	Catalyst concentration (CC)	Yield
59^o^C	9.21	2.96	95.38%

#### Polynomial regression equation.

Yield = 60.2702 + (0.0000 * 1) + (0.2694 * Reaction Temperature) + (2.6898 * Methanol to oil molar) + (10.0242 * Catalyst Concentration) + (−0.0114 * Reaction Temperature^2) + (0.0072 * Reaction Temperature * Methanol to oil molar ratio) + (0.3353 * Reaction Temperature * Catalyst Concentration) + (−0.1406 * Methanol to oil molar ratio^2) + (−0.1714 * Methanol to oil molar ratio * Catalyst Concentration) + (−4.7206 * Catalyst Concentration^2)

## Conclusion

The machine learning validation and optimization study successfully identified the most reliable predictive model and optimal process parameters for biodiesel production.

- Polynomial Regression (PR) demonstrated the highest prediction accuracy, exhibiting strong alignment between experimental and predicted biodiesel yield (R² = 0.956). Its reliability was further validated through k-fold cross-validation, yielding a mean R² score of 0.868, ensuring robust and stable predictions across varying conditions. These findings underscore PR’s effectiveness in capturing complex interactions within biodiesel production systems.
- Catalyst concentration identified as the dominant contributing factor, contributing roughly 95% of the impact on biodiesel yield, as confirmed by SHAP values and feature importance analysis. The Pearson correlation heatmap further reinforced this observation, showing a strong positive correlation (0.93) with biodiesel yield, highlighting the necessity of precise catalyst optimization to maximize conversion efficiency
- Optimization analysis using the PR model successfully identified the optimal process conditions for achieving maximum biodiesel yield (95.38%), with a RT of 59°C, a MOR of 9.21, and a CC of 2.96%. These results emphasize the critical role of fine-tuning process parameters to ensure efficient and stable biodiesel production
- Overall, this study validates the efficacy of PR-based optimization in enlightening biodiesel yield prediction and refining key process conditions, offering a reliable approach for enhancing production efficiency and sustainability
- Although the findings are encouraging, several constraints remain, offering valuable directions for future research and methodological enhancement. The dataset, restricted to 27 experimental data points, may limit predictive generalizability, necessitating expansion to improve model robustness. Additionally, reliance on regression models constrains predictive flexibility, making ensemble learning methods for enhancing predictive stability. Furthermore, incorporating additional input features such as mixing speed, feedstock composition, and reaction time could further refine biodiesel yield optimization. These advancements will improve process efficiency and expand the applications of machine learning in sustainable biodiesel production.

## Abbreviations

ML: Machine Learning
PR: Polynomial Regression
DT: Decision Tree
RF: Random Forest
LR: Linear Regression
NaOH: Sodium Hydroxide
KOH: Potassium Hydroxide
RMSE: Root Mean Square Error
MSE: Mean square Error
MAE: Mean Absolute Error
SHAP: SHapley Additive exPlanations
R2: Coefficent of Determination
RT: Reaction Temperature
CC: Catalyst concentration
MOR: Methanol to oil molar ratio.
